# Risk factors associated with the severity of COVID-19 in a region of the Brazilian Amazon

**DOI:** 10.1038/s41598-021-00009-y

**Published:** 2021-10-18

**Authors:** Daniele Melo Sardinha, Rosane do Socorro Pompeu de Loiola, Ana Lúcia da Silva Ferreira, Carmem Aliandra Freire de Sá, Yan Corrêa Rodrigues, Karla Valéria Batista Lima, Ricardo José de Paula Souza e Guimarães, Luana Nepomuceno Gondim Costa Lima

**Affiliations:** 1 Programa de Pós-Graduação em Epidemiologia e Vigilância em Saúde, Instituto Evandro Chagas (PPGEVS/IEC), Ananindeua, Pará Brazil; 2 Programa de Pós-Graduação em Biologia de Agentes Infecciosos e Parasitários, Universidade Federal do Pará (PPGBAIP/UFPA), Belém, Pará Brazil; 3 Programa de Pós-Graduação em Biologia Parasitária na Amazônia, Universidade do Estado do Pará, Instituto Evandro Chagas (PPGBPA/UEPA/IEC), Ananindeua, Pará Brazil

**Keywords:** Respiratory tract diseases, Public health

## Abstract

The Brazilian Northern region registered a high incidence of COVID-19 cases, particularly in the state of Pará. The present study investigated the risk factors associated with the severity of COVID-19 in a Brazilian Amazon region of 100,819 cases. An epidemiological, cross-sectional, analytical and demographic study, analyzing data on confirmed cases for COVID-19 available at the Brazilian Ministry of Health's surveillance platform, was conducted. Variables such as, municipalities of residence, age, gender, signs and symptoms, comorbidities were included and associated with COVID-19 cases and outcomes. The spatial distribution was performed using the ArcGIS program. A total of 100,819 cases were evaluated. Overall, patients had the mean age of 42.3 years, were female (51.2%) and with lethality reaching 4.79% of cases. Main symptoms included fever (66.5%), cough (61.9%) and sore throat (39.8%). Regarding comorbidities, most of the patients presented cardiovascular disease (5.1%) and diabetes (4.2%). Neurological disease increased risk of death by nearly 15 times, followed by obesity (5.16 times) and immunodeficiency (5.09 time). The municipalities with the highest incidence rate were Parauapebas, Canaã dos Carajás and Jacareacanga. Similarity between the Lower Amazon, Marajó and Southwest mesoregions of Pará state were observed concerning the highest morbidity rates. The obtained data demonstrated that the majority of cases occurred among young adults, females, with the classic influenza symptoms and chronic diseases. Finally, data suggest that the highest incidences were no longer in the metropolitan region of the state. The higher lethality rate than in Brazil may be associated with the greater impacts of the disease in this Amazonian population, or factors associated with fragile epidemiological surveillance in the notification of cases of cure.

## Introduction

The Severe Acute Respiratory Syndrome (SARS) caused by coronavirus-2 (SARS-CoV-2) is an airborne and highly contagious infection, characterized by flu-like symptoms to severe respiratory symptoms, called COVID-19. After a rapidly spread worldwide, the SARS-CoV-2 pandemic has been considered a global emergency due to the significant social-economic and lives losses^1, 2^.

In average 5 days after exposure to the virus, individuals may go asymptomatic or present a wide range of symptoms, including mild to fulminant clinical manifestations, such as fever, dry cough, shortness of breath, pneumonia, muscle disorder characterized by myalgia and Guillain–Barré syndrome^3^. The diagnosis of COVID-19 is usually directly performed through detection of SARS-CoV-2 RNA by reverse-transcription polymerase chain reaction (RT-PCR), or indirectly by means of serologic tests. In addition, clinical abnormalities observed in computed tomography (CT) and laboratory tests, including ground-glass opacities, lymphopenia, elevated lactate dehydrogenase and alteration of the d-dimer may support diagnosis of individuals with a high clinical suspicion of infection^4^.

Mortality on COVID-19 setting is primarily associated with clinical complications, where specific groups more likely to evolve to severe cases, including elderly, pregnant women and those with chronic diseases; and often requiring intensive care, mechanical ventilation, use of antibiotics for secondary infections treatment and invasive maneuvers^5–7^. Deaths worldwide associated with SARS-CoV-2 reached the number of 2,244,213 by February, 2021, of which 1,062,191 registered in the Americas, followed by 759,122 in the European region. In the Americas, the United States and Brazil accounts for the highest number of deaths^8^. In Brazil, community transmission by SARS-CoV-2 was declared on March, 2020, and until February, 2021, 8,447,645 cases and 232,170 deaths were confirmed, with a lethality rate of 2.4%^9^. Among the Brazilian regions, the Northern region stood out with the highest mortality in the country, with a mortality rate of 128.1/100,000 inhabitants on February, 2021^9^. In the region, the state of Pará presented the highest incidence, with 341,05 confirmed cases and 7854 deaths^10^.

The state of Pará have a great territorial extension and present a heterogeneous population composition, which may reflect on particular features and trends of distribution of COVID-19 in the region compared to other locations within the Brazilian territory and worldwide. The present study investigated the risk factors associated with the severity of COVID-19 in a Brazilian Amazon region of 100,819 cases.

## Methods

### Study design and geographical delineation

This is an epidemiological, cross-sectional, analytical and demographic study, which evaluated information on confirmed cases for COVID-19 available at OpenDataSUS (https://opendatasus.saude.gov.br/), a public data platform of the Brazilian Ministry of Health, from March 01 to July 29, 2020.

**Figure 1:**
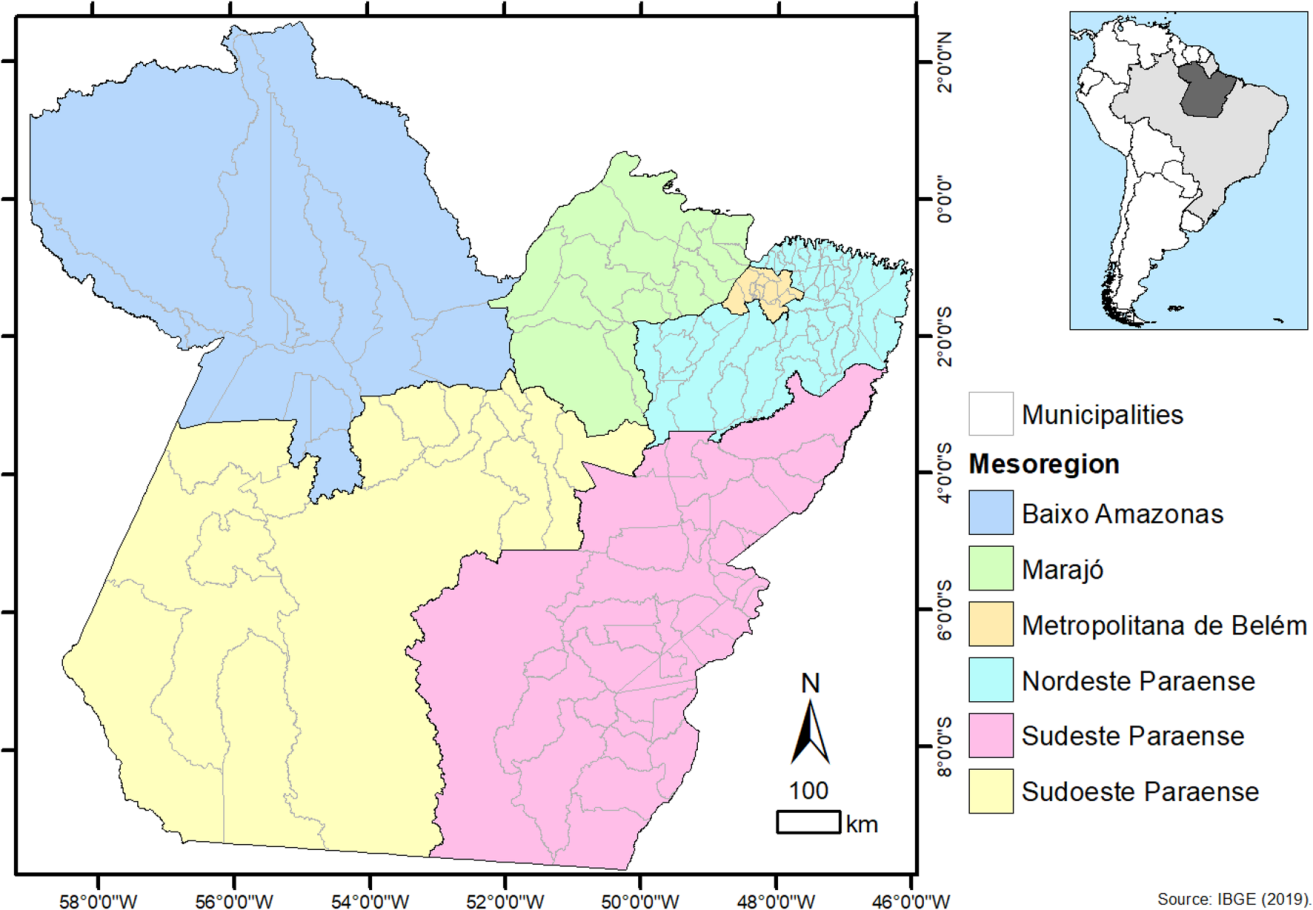
Spatial location of the mesoregions and municipalities of the State of Pará-Amazon-Brazil. Source: OpenDataSUS, Ministry of Health. Software: ArcGis ((https://www.arcgis.com/).

The study included data from the state of Pará, Northern Brazil, the second largest Brazilian state, with a land area of 1,245,870.798 km^2^. The state has six Mesoregions, comprised of 22 Microregions, in a total of 144 municipalities, with the city of Belém as its capital (Fig. 1)^11^. The state of Pará territory is composed by the largest tropical forest in the world, the Amazon Rainforest, with a low and flat relief, and with 58% of the territory is below 200 m, while altitudes above 500 m are in observed in Serra dos Carajás, Serra do Cachimbo and Serra do Acari^12^. The estimated population for 2020 was 8,690,745 inhabitants with a Human Development Index (HDI) of 0.646.

### Data collection and inclusion criteria

The included data was obtained from Brazilian surveillance platforms for COVID-19: The 'E-SUS notifies’, which is exclusive for the notification of suspected cases of respiratory syndrome by COVID-19, and the Epidemiological Surveillance Information System platform of Flu (SIVEP-GRIPE), which carries out the surveillance of all severe acute respiratory syndromes regardless of the etiology. Confirmed cases for COVID-19 and residents of the state of Pará were included, while those with lack of information and/or duplicated by notification register number, were excluded from analysis.

An interface of the two platforms was developed by filtering cases of flu-like illness confirmed to COVID-19 (E-SUS notifies) and cases of SARS confirmed to COVID-19 (SIVEP-GRIPE), generating a total of 89,891 and 12,681 cases from each platform, respectively. Of the 102,572 identified patients, 1753 were excluded due to lack of data, totaling 100,819 cases. Clinical-epidemiological data was extracted and evaluated, including: municipality of residence, age, sex, symptoms, comorbidities, confirmation criteria, cases outcome cases.

### Statistical analysis

Database was constructed into a spreadsheet in Microsoft Excel software for Windows (2019) and statistically analyzed in SPSS 20.0 and MINITAB software. Descriptive analysis was performed using the absolute (Fa) and relative (Fr) frequency distributions of the study variables. Values of p ≤ 0.05 were considered statistically significant.

Association between outcome (dependent) and predictor (independent) variables was assessed by the Chi-square test or Fisher's exact test, when applicable. The lethality rate by age group and overall was performed. The Mann–Whitney test was applied to verify the age differences between sexes, and among survivors and those who evolved to death.

The multiple logistic regression test was applied to assess the association of death outcome and comorbidities, including: diabetes, obesity, asthma, immunodeficiency, heart, lung, neurological, kidney, hematological and liver diseases. Similarly, death outcome was associated with the presence of symptoms, such as: cough, nausea, headache, runny nose, nasal congestion, sore throat, myalgia/arthralgia, diarrhea, chills, adynamia and odynophagia. Receiver operating characteristic (ROC) curve and area under curve (AUC) were calculated and plotted based on statistically significant variables after binary logistic regression analysis, allowing evaluation of sensitivity and specificity of each symptoms/model in predicting the severity for COVID-19.

The morbidity index (MI) strategy was applied by transforming qualitative variables into decimal base numbers to assess the distribution and contribution of comorbidities to death outcome across the different mesoregions within State of Pará. An information grid arranging variables with a higher odds ratio for death outcome was constructed according to the following mathematical model Morbidity Index (IM) $={\sum 2}^{n}.X;$ where n represents morbidity and X the presence (1) or absence (0)^13^. Kruskal–Wallis test was applied to verify the distribution of MIs among survivors and those who evolved to death, and also to verify their distribution among survivors and those who evolved to death within the mesoregions of State of Pará.

Pearson's correlation was applied to verify the association of age and clinical outcome, as well as with comorbidities and geographic mesoregions of State of Pará. Multivariate analysis using the simple Euclidean distance aggregation method was applied to assess similarities among mesoregions State of Pará according to the presence of comorbidities.

### Spatial analysis

The cartographic bases used were obtained from the Brazilian Institute of Geography and Statistics (http://www.ibge.gov.br/). The study area and spatial distribution of cases in the municipalities of Pará were performed in ArcGIS software (https://www.arcgis.com/) and classified according to quartile into classes, based on the calculation of incidence (number of cases/population X 100,000): quartile 1 (124.3–623.5), quartile 2 (623.6–1142.6), quartile 3 (1142.7–2002.5), quartile 4 (2002.6–3608.6), quartile 5 (3608.7–5458.8). The municipalities with quartile 5 were named on the map.

### Ethical considerations

The present study evaluated coded secondary data, without any health risk and possibility of identifying the respective patients, and was conducted in accordance to the Brazilian Law No. 12.527 of 18/11/2011, which regulates access to information^14^.

## Results

**Figure 2:**
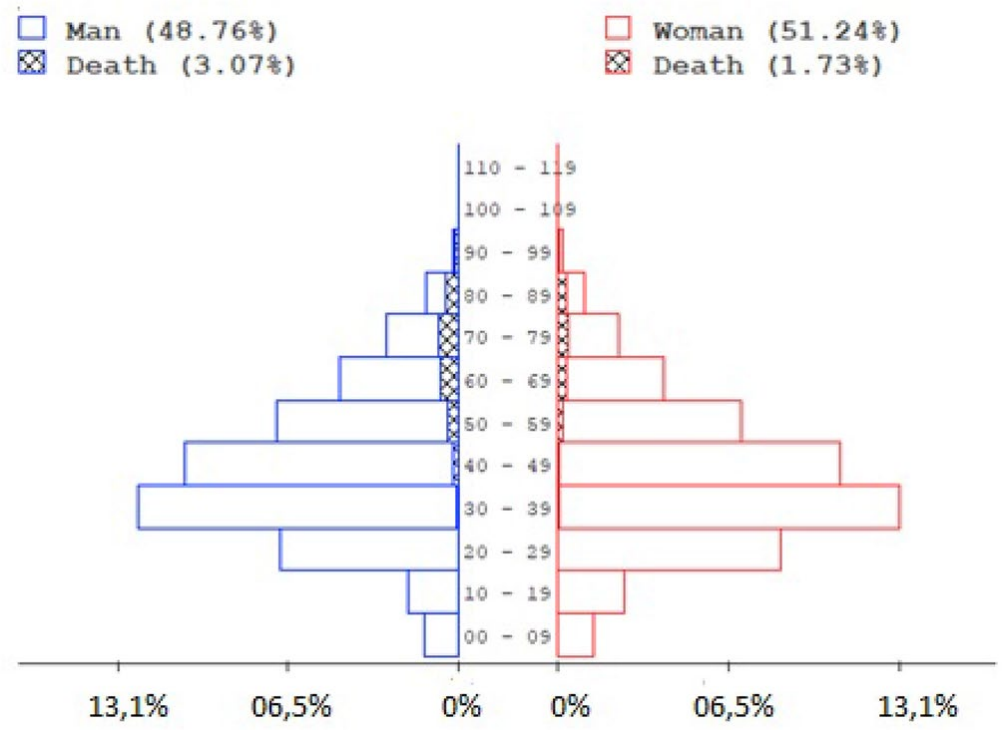
Distribution by sex, age, and clinical outcome of cases of SARS-CoV-2 infection in the State of Pará-Amazon-Brazil, 2020. Source: OpenDataSUS, Ministry of Health.

From the total of 100,819 evaluated cases, 71,500 (70.9%) were confirmed by rapid test, 21,056 (20.9%) by RT-PCR, and 8263 (8.2%) by clinical-epidemiological criteria. Patients’ mean age was 42.30 ± 17.60 years, and those within the age range of 20 and 60 years, comprising 76.25% (76,877/100,819) presented higher susceptibility to the disease (χ^2^ = 43,878.19; p < 0.0001). Regarding gender, 51.24% were women (51,661/100,819) and 48.76% (49,158/100,819) were men, with the proportion of affected women significantly differing compared to men (χ^2^ = 62.141; p < 0.0001) (Fig. 2).

Table 1 presents the distribution of the clinical-epidemiological variables included in this analysis. The most frequent symptoms were fever (67,012/66.5%), cough (62,438/61.9%), sore throat (40,138/39.8%), dyspnea (34,862/34.6%), headache (24,671/24.5%) and myalgia/arthralgia (17,963/17.8%). Regarding the presence of comorbidities, 9.72% of patients presented at least one comorbidity, and of these, 2.1% had two or more associated conditions. The most common diseases included cardiovascular diseases and diabetes, representing 5.06% and 4.20% of the cases, respectively (Table 1). Considering all evaluated comorbidities, the obtained MI ranged from 0 to 1023 with a mean of 6.91 ± 34.37 among the 100,819 individuals. Among survivors, it ranged from 0 to 1023 with a mean of 5.17 ± 27.43, and among those who died, ranging from 0 to 872, with a mean of 41.44 ± 91.84.

**Table 1:** Clinical characteristics of SARS-CoV-2 infected people in a region of the Brazilian Amazon, 2020. Source: OpenDataSUS, Ministry of Health.

Characteristics	Total of cases (N = 100,819)		Survival (N = 95,984)		Dead (N = 4835)		p-value
	N	%	N	%	N	%	
**Symptoms**							
Fever	67,012	66.5	63,340	65.99	3672	75.95	< 0.0001
Cough	62,438	61.9	58,694	61.15	3744	77.44	< 0.0001
Pharyngalgia	40,138	39.8	38,861	40.49	1277	26.41	< 0.0001
Dyspnea	34,862	34.6	30,833	32.12	4029	83.33	< 0.0001
Headache	24,671	24.5	24,313	25.33	358	7.4	< 0.0001
Myalgia/arthralgya	17,963	17.8	17,555	18.29	408	8.44	< 0.0001
Diarrhea	9806	9.7	9383	9.78	423	8.75	0.02
Coryza	8603	8.5	8475	8.83	128	2.65	< 0.0001
Adynamia	4244	4.2	4099	4.27	145	3	< 0.0001
Nausea	3859	3.8	3598	3.75	261	5.4	< 0.0001
Chills	2342	2.3	2281	2.38	61	1.26	< 0.0001
Nasal congestion	1955	1.9	1881	1.96	74	1.53	0.0396
Odinophagy	963	1	930	0.97	33	0.68	0.0546
Conjunctivitis	199	0.2	191	0.2	8	0.17	0.7289
**Comorbidities**							
Cardiovascular disease	5106	5.1	3963	4.13	1143	23.64	< 0.0001
Diabetes	4236	4.2	3211	3.35	1025	21.2	< 0.0001
Asthma	721	0.7	684	0.71	37	0.77	0.7366
Pulmonary disease	583	0.6	439	0.46	144	2.98	< 0.0001
Kidney disease	534	0.5	379	0.39	155	3.21	< 0.0001
Immunodeficiencies	442	0.4	328	0.34	114	2.36	< 0.0001
Obesity	365	0.4	241	0.25	124	2.56	< 0.0001
Neurological disease	186	0.2	92	0.1	94	1.94	< 0.0001
Hematological disease	56	0.1	42	0.04	14	0.29	< 0.0001
Liver disease	56	0.1	42	0.04	14	0.29	< 0.0001

*G test.

### Risk of death

**Figure 3:**
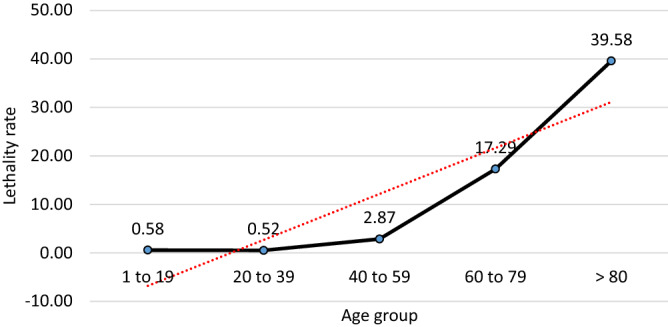
Fatality rate by age group of cases of SARS-CoV-2 infection in the State of Pará-Amazon-Brazil, 2020. Source: OpenDataSUS, Ministry of Health.

Among the investigated cases, 4.79% (4835/100,819) had death as outcome, which mostly occurred among men (63.97%—3093/4835), indicating a twice higher relative risk of death when compared to than women (36.03%—1742/4835) (Odds Ratio 1.92; p < 0.001; 95% CI 1.82 ≤ µ ≤ 2.04) (Fig. 2). In the lethality rate by age group, > 80 accounted for (39.58%) lethality, followed by 60–79 (17.29%), 40–59 (2.87%), 20–39 (0.52%) and 1–19 years (0.58%) (Fig. 3). Risk of death was associated with age, as demonstrated by Pearson's test, which revealed a positive and significant correlation between death and advanced age (r = 0.323; p < 0.001) patients, and by Mann–Whitney test, which associated the median age of 70 years to death outcome (p < 0.0001).

The presence of comorbidities among survivors (8.1%—7762/95,984) and those who evolved to death (42.2%—2040/4835) significantly differed as verified by the binomial Chi-square test (95% CI 40.8–43.6; p < 0.0001). All comorbidities were associated with mortality of patients, except asthma (0.8%) ([95% CI 0.6–1.1]; p = 0.7366) (Tables 1, 2).

**Table 2:** Predicting variables of risk for death in SARS-CoV-2 infection in the population of the State of Pará-Amazônia-Brazil, 2020. Source: OpenDataSUS, Ministry of Health.

Variables	p-value	Odds ration	95% CI for Odds ratio	
			Inferior	Upper
**Symptoms**^a^				
Dyspnea	< 0.0001	7.766	7.137	8.45
Nasal congestion	< 0.0001	1.859	1.398	2.472
Gender	< 0.0001	1.784	1.664	1.914
Nausea	< 0.0001	1.611	1.375	1.888
Cough	< 0.0001	1.233	1.132	1.343
Fever	< 0.0001	1.196	1.1	1.301
Age	< 0.0001	1.082	1.079	1.084
Adynamia	0.005	0.761	0.629	0.922
Myalgia/arthralgya	< 0.0001	0.619	0.55	0.696
Coryza	< 0.0001	0.586	0.479	0.717
Pharyngalgia	< 0.0001	0.544	0.503	0.588
Headache	< 0.0001	0.386	0.342	0.436
**Comorbities**^b^				
Neurological disease	< 0.0001	14.442	10.458	19.943
Obesity	< 0.0001	5.139	3.987	6.624
Immunodeficiency	< 0.0001	5.023	3.937	6.409
Diabetes	< 0.0001	4.214	3.855	4.606
Cardiovascular disease	< 0.0001	4.090	3.759	4.450
Pulmonary disease	< 0.0001	3.368	2.700	4.201
Kidney disease	< 0.0001	3.234	2.594	4.034

Logistic Regression model: ^a^(χ^2^(12) = 14,646,45; p-value < 0.0001; R^2^ Nagelkerke = 0.423), ^b^(χ^2^(7) = 3834,89; p-value < 0.0001; R^2^ Nagelkerke = 0.117).

In addition, the binary logistic regression model with death as the dependent variable and symptoms as predictors was significant (χ^2^(12) = 14,646.45; p < 0.001; R2 Nagelkerke = 0.423). Variables were associated with death, included age, sex, fever, cough, dyspnea, nausea, and nasal congestion; while headache, myalgia, runny nose, adynamia and sore throat were associated with survival (Table 2). According to this model, death among patients who reported dyspnea increased by 676.6%; in the same way as the presence of nasal congestion, nausea and being male and feeling nausea, the increase in death was around 85.9%, 61.1% and 78.4%, respectively.

**Figure 4:**
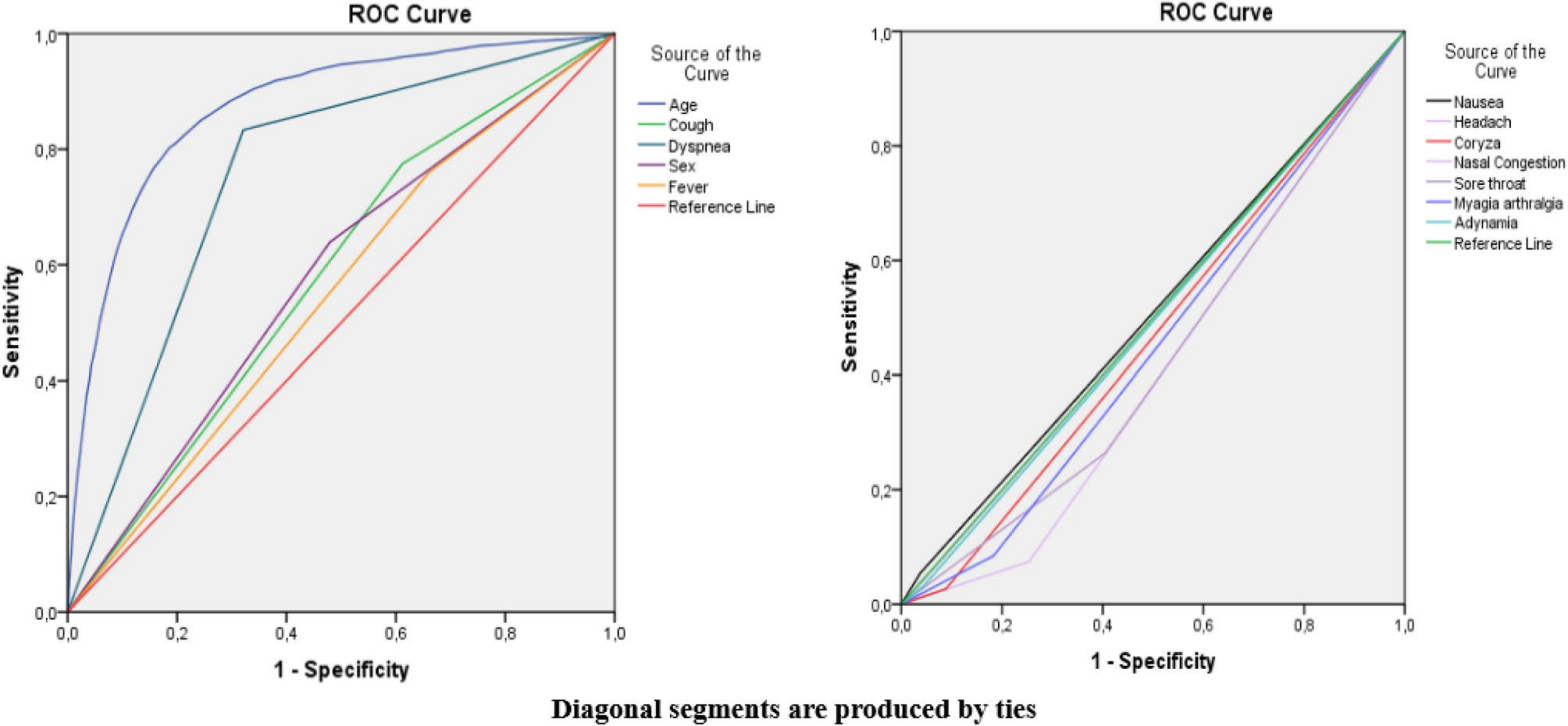
ROC curves of age, dyspnea, cough, sex, fever, nausea, nasal congestion, adynamia, coryza, myalgia, arthralgia, sore throat, headache, and logistic regression model in patients with COVID-19. Source: OpenDataSUS, Ministry of Health.

The ROC curves are presented in Fig. 4. Since the platelet was a protective factor, all patients’ platelet values were multiplied by − 1 to make its ROC curve above the reference line. The above parameters were all valuable for predicting the severity of COVID-19 (*p* < 0.05). The AUC of the logistic regression model was 0.774 (95% CI 0.722–0.827). The AUC and optimal thresholds of each independent risk or protection factors are presented in Table 3.

**Table 3:** The AUC of each symptom associated with the clinical outcome of COVID-19. Source: OpenDataSUS, Ministry of Health.

Variables	AUC	Std. error^a^	*p*-value	IC: 95% confidence interval	
				Lower bound	Upper bound
Age	0.873	0.003	< 0.0001	0.868	0.879
Dyspnea	0.756	0.003	< 0.0001	0.750	0.762
Cough	0.581	0.004	< 0.0001	0.574	0.589
Sex	0.580	0.004	< 0.0001	0.572	0.588
Fever	0.550	0.004	< 0.0001	0.542	0.558
Nausea	0.508	0.004	0.053	0.500	0.517
Nasal_congestion	0.498	0.004	0.614	0.490	0.506
Adynamia	0.494	0.004	0.135	0.485	0.502
Coryza	0.469	0.004	< 0.0001	0.461	0..477
Myalgia arthralgia	0.451	0.004	< 0.0001	0.443	0.458
Sore throat	0.430	0.004	< 0.0001	0.422	0.438
Headache	0.410	0.004	< 0.0001	0.403	0.418

In further analysis, the presence of comorbidities was associated with risk of death among patients, including neurological disease, obesity, immunodeficiency, diabetes, cardiovascular diseases, Pneumopathy, Nephropathy, asthma e liver diseases in the binary logistic regression model. Hematologic disease was excluded from the model due to its high collinearity, as well as, liver disease and asthma due to lack of significancy in the model. Thus, logistic regression was significant (χ^2^(7) = 3834,89; p < 0.0001; R^2^ Nagelkerke = 0.117), revealing that the risk of death among patients reporting neurological diseases increased 14.48-fold, followed by obesity (odds ratio 5.16) and immunodeficiency (odds ratio 5.09) (Table 2). Death increased by 1341.9% among patients with neurological diseases, followed by obesity (413.9%) and immunodeficiency (402.3%).

**Figure 5:**
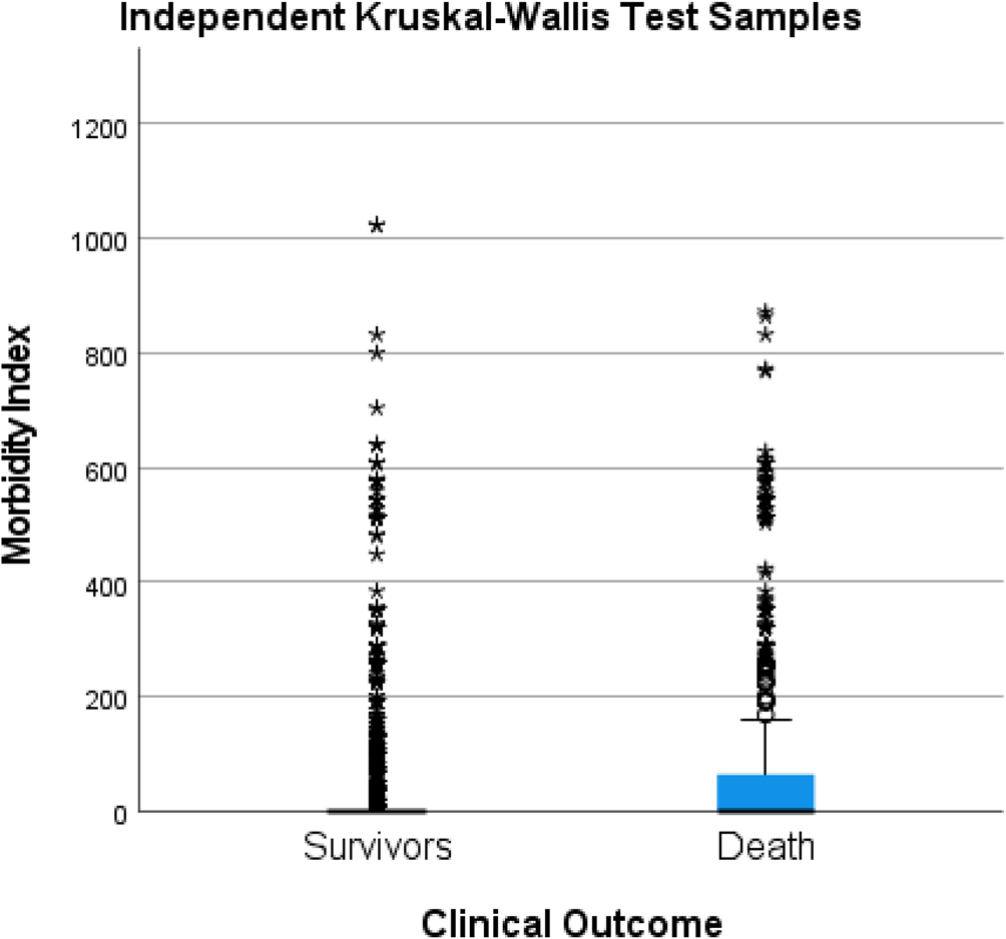
Comparison of the morbidity rate between the clinical outcomes, survivor and death, of cases of SARS-CoV-2 infection in the State of Pará-Amazon-Brazil, 2020. Source: OpenDataSUS, Ministry of Health.

Finally, the distribution of the mean MIs among survivors and those who died revealed a highly significant difference by the Kruskal–Wallis test (H = 6351.263; GL = 1; p-value < 0.001) (Fig. 5).

### Spatial analysis

The presence of comorbidities was not associated with any of the geographic mesoregions of the State of Pará, as verified Pearson's test. The distribution of MIs between the different geographic mesoregions did not differ by the Kruskal–Wallis independence test among those who progressed to death (χ^2^ = 7.967; gl = 5; p = 0.158), however, when considering survivors, the MIs significantly differed (χ^2^ = 779.546076; gl = 5; p-value < 0.0001) (Table 4). Given the statistical significance of the test applied, we followed the test of comparison between the morbidity rates of the mesoregions by the Pairwise Method with Bonferroni correction, revealing statistically significant differences, as presented in Table 4. In this aspect, it was verified that the mean ranks of the MI in the Southeast mesoregion were lower than in the Southwest, but higher compared to Lower Amazon, Marajó, and Northeast mesoregions. Regarding Southwest mesoregion, the mean ranks of the MI were higher than Marajó, Belém metropolitan region and Northeast mesoregions. In Lower Amazon mesoregions, mean ranks of the MI were lower concerning that of observed in Belém Metropolitan region, Marajó, and Northeast mesoregions.

**Table 4:** Distribution of the morbidity rate in relation to the clinical outcome facing infection by SARS-CoV-2 in the geographic regions of the State of Pará-Amazon-Brazil, 2020. Source: OpenDataSUS, Ministry of Health.

Geographical region Para State	Morbidity index		Morbidity index comparison of the survivors	
	Survivor	Death	Region	p-value
**Lower Amazon**^**1**^				
N	6728	328	5–6	< 0.0001
Min–Max	0–832	0–528	5–1	< 0.0001
Average	4.32 ± 24.32	39.52 ± 67.30	5–2	< 0.0001
**Marajó**^**2**^			5–3	< 0.0001
N	5178	213	5–4	< 0.0001
Min–Max	0–512	0–628	6–1	1.000
Average	5.38 ± 26.35	38.89 ± 99.03	6–2	< 0.0001
**Metropolitan**^**3**^			6–3	< 0.0001
N	26,312	2616	6–4	< 0.0001
Min–Max	0–800	0–864	1–2	< 0.0001
Average	7.07 ± 34.27	44.81 ± 100.65	1–3	< 0.0001
**Northeast**^**4**^			1–4	< 0.0001
N	23,909	910	2–3	1.000
Min–Max	0–1023	0–832	2–4	0.512
Average	6.05 ± 27.03	34.86 ± 77.27	3–4	1.000
**Southeast**^**5**^				
N	26,078	599		
Min–Max	0–1023	0–608		
Average	3.03 ± 21.77	39.53 ± 80.32		
**Southwest**^**6**^				
N	7779	169		
Min–Max	0–512	0–872		
Average	3.75 ± 21.81	38.44 ± 90.15		

**Figure 6:**
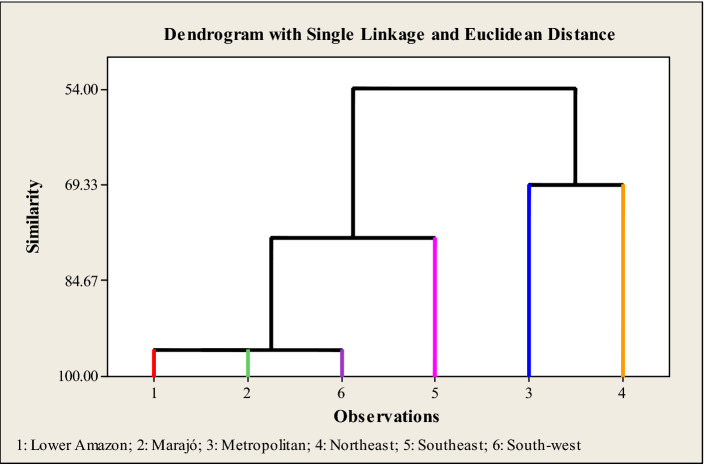
Similarity of geographical regions according to the presence of morbidities in individuals with SARS-CoV-2 infection registered from the State of Pará-Amazonia-Brazil, 2020. Source: OpenDataSUS, Ministry of Health.

Also, considering the Kruskal–Wallis statistical differences between groups, this study tested whether the presence of the comorbidities clustered with the mesoregions. Thus, multivariate analysis by the simple clustering method using the Euclidean distance without standardization revealed similarity, which ranged from 54.00 to 95.77% among three different mesoregions groups, where similarity was observed between the mesoregions of Lower Amazon, Marajó and Southwest, as presented in Fig. 6.

**Figure 7:**
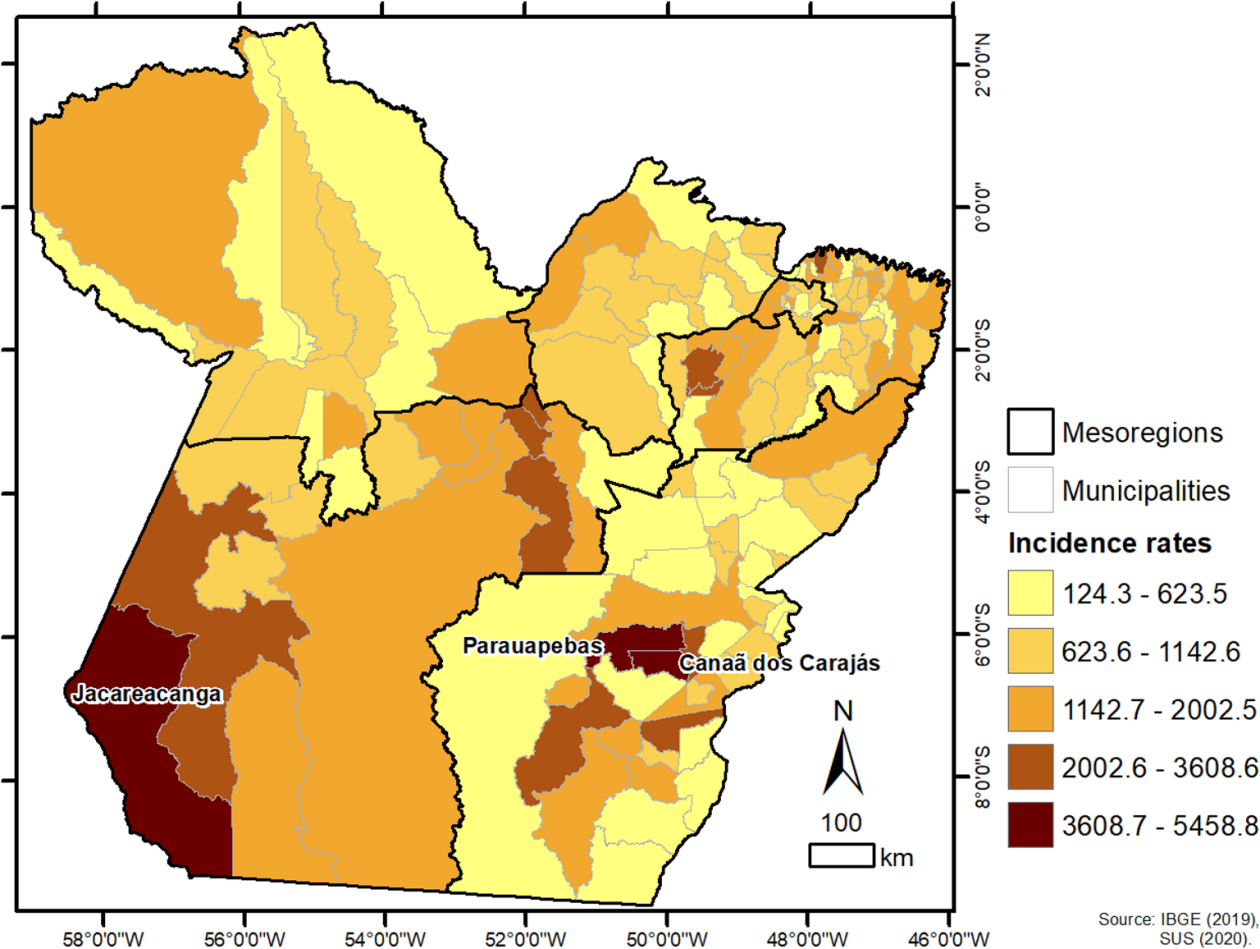
Spatial distribution of the incidence of cases of COVID-19 by municipalities of the State of Pará-Amazon-Brazil, in the period from March to June/2020. Source: OpenDataSUS, Ministry of Health. Software: ArcGis ((https://www.arcgis.com/).

**Figure 8:**
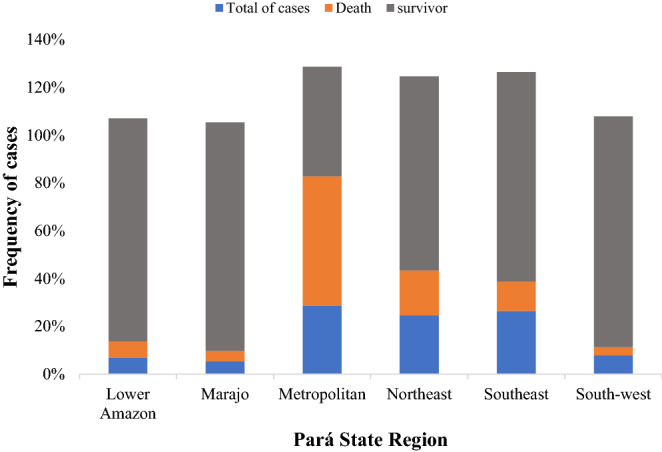
Frequency of cases of SARS-CoV-2 infection distributed by clinical outcomes and regions of the State of Pará-Amazonia-Brazil, 2020. Source: OpenDataSUS, Ministry of Health.

The spatial distribution of COVID-19 cases was heterogenous within Pará state, being possible to observe areas concentrating higher incidence rates, compared to lower rates in other areas (Fig. 7). The incidence of the disease ranged from 124.3 to 5458.8, with an average of 1091.89. The highest incidence was registered in the Southwest mesoregion, with 1488.49, ranging from 530.84 to 5256.92, with a mean of 1756.62 and standard deviation of 1344.89. Metropolitan region of Belém registered most of infection cases (Fig. 8), with an incidence of 1059.86, ranging from 396.26 to 2002.57, mean 913.75 and standard deviation 465.47. Most cases diagnosed in Pará State, about 87.90% (88,618/100,819), were due to local transmission.

## Discussion

The present study evaluated the clinical-epidemiological and spatial factors associated with 100,819 cases confirmed for COVID-19 in the State of Pará, the second-largest Brazilian, located in the Brazilian Amazon region. In this study, the case fatality rate was 4.8%, which is higher than the rate reported in Brazil for the same study period, which was (3.1%)^15^.

AUC revealed that age, dyspnea, cough, gender and fever were considered predictors to the severity of COVID-19. Salzberger et al.^16^ describe that 5–10% of patients with SARS poorly progress, leading to a mortality rates up to 1.4%, especially among those with 60 years of age or more. The obtained data showed that the mean age of patients was 42.3 years and mostly females (51.2%), but with most of death cases occurring among men, and with and a median age of 70 years, similarly as observed in Beijing, China^17, 18^.

Ambrosino et al.^19^ highlight that age above 60 years is a risk factor for mortality and can be aggravated by the presence of chronic diseases such as hypertension, diabetes, cardiovascular diseases, hypercholesterolemia and obesity conditions. In addition, elderly patients become more susceptible to the severity of SARS-CoV-2 infection due to use of polypharmacy and deficient immune response associated with immunosenescence^20^. On the other hand, a dysregulated immune response may lead to atypical clinical presentations, such as the absence of fever, which is the main sign of infection, impairing adequate screening for the disease in this age group^21^. Finally, the most reported symptoms among this population are in line with observations from several studies^22–25^.

Regarding comorbidities, 9.7% of patients presented at least one comorbidity, while 2.1% had two or more comorbidities. Among survivors, 8.1% had at least one comorbidity, and 42.2% among those who died also had one, the most common being cardiovascular diseases (5.1%) and diabetes (4.2%). However, when it comes to the odds ratio of dying, neurological diseases represented the comorbidity as the main predictor of mortality by (14.48) times, followed by obesity (5.16) and immunodeficiency (5.09).

As highlighted by several studies, conditions such as smoking, diabetes, and hypertension, which are even related to disease severity, increase ACE2 expressions, enzyme which its higher expression may increase vulnerability to SARS-CoV-2. Additionally, ACE2 expression is higher in cardiac and pulmonary tissues, which contributes to severe pulmonary and cardiovascular complications. Finally, a hyperinflammatory response leads to a drop in O_2_ saturation and complications in myocardial stability, leading to cardiopulmonary failures, such as acute cardiac injury and ground-glass lung lesions on CT scans, which are serious complications directly associated with mortality^26–29^.

Studies from New York and the United Kingdom show a higher prevalence of comorbidities than this study^30, 31^ but are similar to the results on comorbidities in patients with COVID-19 in a meta-analysis and the COVID-19 Brazil Bulletin^17, 32^. The presence of comorbidities, especially cardiovascular disease, diabetes, and kidney disease, is a risk factor for mortality, so specific strategies should be directed to this group to reduce deaths^33^.

Regarding neurological diseases as a high predictor for mortality, García-Azorín et al.^34^ showed in a retrospective cohort of those hospitalized for COVID-19, that the presence of neurological diseases is the main independent predictor for COVID-19 mortality among older people and those with other risk factors, such as cardiovascular.

In the present study, obesity increased the risk of death by 5 times. A study conducted in three hospitals in China, with obese patients (75) and non-obese controls (75), showed that obese patients were admitted to hospital with high C-reactive protein and low lymphocytes compared to controls, leading to a longer hospital stay and a three times higher risk of severe COVID-19. Authors suggest that the underlying chronic low-grade inflammation and suppression of innate and adaptive immune responses are associated with poor outcomes among obese patients, as well as that obesity may affect mechanical dysfunction, which is a factor for lower respiratory tract infection severity and secondary infections^35^.

In line with previous reports, immunodeficiency was a major risk factor for death in the present study, increasing its chances by five times. The meta-analysis of Gao et al.^36^ showed that immunosuppressed patients had 3.39 times higher chances of poor clinical evolution and mortality. To Babaha e Rezaei^37^, unfavorable outcomes in COVID-19 setting are related to opportunistic and/or secondary infections, specially by bacterial and fungal agents. By contrast, immunosuppression causing B-lymphocyte deficiency may minimize the effects of the inflammatory cytokines storm, leading to mild symptoms cases. Thus, B-lymphocyte deficiency may prevent the hyper-inflammation process, however, predisposes patients to other potentially fatal infections.

Our data evidenced that all comorbidities, except asthma, were significantly related to patients’ mortality. Although the World Health Organization (WHO) and the United States Centers for Disease Control and Prevention (CDC) include asthma patients as risk groups for COVID-19, asthma patients accounted only for 12% of COVID-19 hospitalizations, a smaller number than the current 20% of asthma patients hospitalized due to influenza in the US. Although inaccuracy on incidence data, studies suggests that asthmatics do not appear to be as affected as other COVID-19 risk groups^28, 38^. Differently from asthma, lung disease was a comorbidity significantly associated with death cases. Early reports also demonstrated that this condition is associated with increased ACE2 expression in lung tissue and small airways, increasing risk for severe clinical manifestations among hospitalized patients for was found in other studies, in which, having lung disease increased the risk of severe COVID-19^28, 38–41^.

In the spatial distribution, the high incidences of COVID-19 in the municipalities of Parauapebas, Canaã dos Carajás and Jacareacanga were concerning. Emergency situation was declared in Jacareacanga city due to lack of health professionals, equipment and medications for treatment of indigenous people in nearby villages, causing high morbimortality among this population and death of at least six indigenous leaders. Additionally, Jacareacanga was among the 10 cities in Brazil with the highest incidence and mortality by COVID-19, with 15.7% of all city population been infected by SARS-CoV-2 by august, 2020^42^.

Regarding the MIs by mesoregion, similarities between the Lower Amazon, Marajó, Southwest mesoregions were observed. The Metropolitan region of Belém presented similarities with the Northeast mesoregion, but with lower MIs compared to others regions. Thus, the higher presence of morbidity in a mesoregion may be associated with distinct characteristics of that population, as well as exposure to environmental factors, resulting in higher rates of infection and mortality due to COVID-19. The clustering similarities between Marajó, Lower Amazon and Southwest mesoregions may be explained by the fact these communities are characterized by a relatively small and fragmented population, strong indigenous component, and poor socioeconomic conditions, factors which could enhance probability of poor prognosis of patients. Finally, the impact of infectious diseases on these groups is not necessarily due to the absence of specific genes related to immune response capacity, but to the fact that these populations, especially the indigenous ones, are biologically very homogeneous from the genetic point of view and lack a social structure capable of providing basic healthcare^43^.

Several mesoregions of Pará suffer from environmental contamination by mercury, with riverside and traditional communities being the most affected. Most of these communities are based along the Amazon River Basin, which permanently receive tons of mercury from illegal gold mining, specially Tapajós River, causing damage to agriculture and fishing actives^44^ A systematic review by Castro and Lima^45^ showed that the riverside populations along the Tapajós River presented Hg levels above the WHO permitted limit. Mercury reaches individuals through local food, the main one being the consumption of fish, which are contaminated during the flow of matter in the food chain. This exposure and intoxication can directly affect the central nervous system and immune system, thus these exposed populations would have more comorbidities than other populations from regions without mining^44^, and this would corroborate the unfavorable outcome of SARS-CoV-2 infection observed in this study, particularly among people with neurological morbidities.

A limitation of this study was the lack of proper filling out the notification forms, causing several variables to be excluded. It is also noteworthy that the epidemiological situation of COVID-19 in this region may be more incident due to several municipalities lacking mass testing capacity or due to underreporting. Another important point to highlight in the limitation is the priority that municipalities perform and investigate the deaths by COVID-19, consequently being the first to be entered into the surveillance system when compared to cases that evolved to the cure, thus many cases of healing have not yet been to the surveillance system which may be associated with higher lethality in this study, the data are subject to change because the cases and deaths are under constant investigations to qualify the data. This limitation is directly associated with the quality of epidemiological surveillance and was experienced by the main author of this study, by several factors, lack of trained professionals and high demand for service.

## Conclusion

The clinical and epidemiological features of COVID-19 in the state of Pará were similar to those in Brazil and other countries. Most infected were female, young adults, with the classic clinical picture of fever, cough, sore throat, myalgia/arthralgia, headache. Most common comorbidities were cardiovascular diseases and diabetes in the study population. However, the characteristics of the deaths identified a higher lethality rate than in Brazil, being male and elderly, with one or more chronic diseases, who presented with dyspnea, nasal congestion, fever, nausea and cough. All comorbidities were associated with deaths, except asthma, which proved not to be a risk factor for complications or deaths for COVID-19. Besides, neurological diseases, obesity, and immunodeficiency stood out in the increased chance ratio of progression to death. The higher lethality rate than in Brazil may be associated with the greater impacts of the disease in this Amazonian population, or factors associated with fragile epidemiological surveillance in the notification of cases of cure.

Furthermore, it was shown that the mesoregions with the highest incidences in the study period were the Southwest and Southeast, which have similar characteristics, such as indigenous populations and mining regions, which require attention from public health policies for these vulnerable populations. Also, we verified being elderly or suffering from chronic diseases and living in certain geographical areas of the state of Pará, such as the Southwest, Southeast and Marajó mesoregions, seem to be predictors for complications and mortality. Finally, we highlight the need of further studies to clarify which risk factors/markers contribute to the severity of COVID-19, which may aid on the development and implementation of surveillance strategies, and reducing mortality within this region.
